# Comparative Genomic Analysis of the Human Pathogen *Wohlfahrtiimonas Chitiniclastica* Provides Insight Into the Identification of Antimicrobial Resistance Genotypes and Potential Virulence Traits

**DOI:** 10.3389/fcimb.2022.912427

**Published:** 2022-07-07

**Authors:** Anna Kopf, Boyke Bunk, Sina M. Coldewey, Florian Gunzer, Thomas Riedel, Percy Schröttner

**Affiliations:** ^1^ Medical Microbiology and Virology, University Hospital Carl Gustav Carus, Dresden, Germany; ^2^ Clinic for Hematology and Oncology, Carl-Thiem-Klinikum, Cottbus, Germany; ^3^ German Collection of Microorganisms and Cell Cultures GmbH, Leibniz Institute DSMZ, Braunschweig, Germany; ^4^ Department of Anesthesiology and Intensive Care Medicine, Jena University Hospital, Jena, Germany; ^5^ Septomics Research Center, Jena University Hospital, Jena, Germany; ^6^ Department of Hospital Infection Control, University Hospital Carl Gustav Carus, Dresden, Germany; ^7^ German Center for Infection Research (DZIF), Partner Site Hannover-Braunschweig, Braunschweig, Germany

**Keywords:** *W. chitiniclastica*, pan-genome, antimicrobial resistance, CRISPR, Acr *(anti-CRISPR)*, *rpoB*, arsenic resistance

## Abstract

Recent studies suggest that *Wohlfahrtiimonas chitiniclastica* may be the cause of several diseases in humans including sepsis and bacteremia making the bacterium as a previously underappreciated human pathogen. However, very little is known about the pathogenicity and genetic potential of *W. chitiniclastica*; therefore, it is necessary to conduct systematic studies to gain a deeper understanding of its virulence characteristics and treatment options. In this study, the entire genetic repertoire of all publicly available *W. chitiniclastica* genomes was examined including *in silico* characterization of bacteriophage content, antibiotic resistome, and putative virulence profile. The pan-genome of *W. chitiniclastica* comprises 3819 genes with 1622 core genes (43%) indicating a putative metabolic conserved species. Furthermore, *in silico* analysis indicated presumed resistome expansion as defined by the presence of genome-encoded transposons and bacteriophages. While macrolide resistance genes *macA* and *macB* are located within the core genome, additional antimicrobial resistance genotypes for tetracycline (*tetH, tetB*, and *tetD*), aminoglycosides (*ant(2’’)-Ia, aac(6’)-Ia*,*aph(3’’)-Ib*, *aph(3’)-Ia*, and *aph(6)-Id*)), sulfonamide (*sul2*), streptomycin (*strA*), chloramphenicol (*cat3*), and beta-lactamase (*blaVEB*) are distributed among the accessory genome. Notably, our data indicate that the type strain DSM 18708^T^ does not encode any additional clinically relevant antibiotic resistance genes, whereas drug resistance is increasing within the *W. chitiniclastica* clade. This trend should be monitored with caution. To the best of our knowledge, this is the first comprehensive genome analysis of this species, providing new insights into the genome of this opportunistic human pathogen.

## Introduction

*Wohlfahrtiimonas chitiniclastica* was first isolated from the larvae of *Wohlfahrtia magnifica* (Tóth et al., 2008), an obligate parasitic fly that causes myiasis by depositing eggs and larvae in mammalian wounds in both animals and humans (Robbins and Khachemoune, 2010). *W. chitiniclastica* are Gram-negative, strictly aerobic, non-motile rods. A key feature is strong chitinase activity, which may be an indicator of a symbiotic relationship with its host fly while also playing an important role in metamorphosis (Tóth et al., 2008; Schröttner et al., 2017; Snyder et al., 2020). Apart from the close association between *W. chitiniclastica* and certain flies (Tóth et al., 2008; Cao et al., 2013; Maleki-Ravasan et al., 2020), the bacteria have recently been detected in various habitats around the world such as arsenic-affected soils from Bangladesh (Sanyal et al., 2016), chicken meat purchased in Brazilian supermarkets (Matos et al., 2019), the pancreas of a Zebra in China (Zhou et al., 2016), and human soft tissue infection in Estonia (Kõljalg et al., 2015), to name but a few. Finally, yet importantly, recent studies indicate that *W. chitiniclastica* can be the cause of several diseases in animals (Thaiwong et al., 2014; Diaz-Delgado et al., 2015; Qi et al., 2016) and humans, including sepsis and bacteremia (Almuzara et al., 2011; Kõljalg et al., 2015; Campisi et al., 2015; Suryalatha et al., 2015), making the bacterium a previously underestimated human pathogen (Schröttner et al., 2017). Although the pathogenesis of *W. chitiniclastica* has not been fully elucidated, the bacterium is thought to invade traumatic skin lesions via fly larvae, resulting in severe myiasis and/or wound contamination (Robbins and Khachemoune, 2010; Thaiwong et al., 2014; Schröttner et al., 2017). However, since most clinicians are unfamiliar with this species and conventional methods often lead to misidentification (Kõljalg et al., 2015; Kopf et al., 2021), it can be assumed that *W. chitiniclastica* has been poorly recognized as a possible cause and is even more common than originally thought (Kopf et al., 2021).

To date, NCBI lists 26 genomes of *W. chitiniclastica* strains, 22 of which have been isolated in the course of human disease. In addition, three draft genomes of strains isolated from an animal source have been published (Cao et al., 2013; Zhou et al., 2016; Matos et al., 2019), and annotations revealed genes encoding for macrolide-specific efflux pumps (*macA* and *macB*) (Matos et al., 2019), a *bla*_VEB-1_ gene cassette, which confers resistance to ceftazidime, ampicillin, and tetracycline (Zhou et al., 2016), and a genome-encoded 25.9kb intact phage (Matos et al., 2019). However, apart from these preliminary genomic studies, very little is known about the genetic potential of *W. chitiniclastica*, making it necessary to initiate systematic studies in order to gain more insight into its virulence characteristics as well as treatment options. Therefore, in this study, the entire genetic repertoire of all publicly available *W. chitiniclastica* genomes was investigated; including *in silico* characterization of the antibiotic resistome, prophage content, and virulence potential. In addition, we performed a pan-genome analysis to elucidate the major genome features and genetic variability of *W. chitiniclastica*. To the best of our knowledge, this is the first comprehensive genome analysis of *W. chitiniclastica* and allows us to better understand this previously underestimated human pathogen.

## Materials and Methods

### Genomic Strain Collection

All publicly available *W. chitiniclastica* genomes were included in this study (n = 26). These include a total of 14 *W. chitiniclastica* isolates from Dresden (Germany) that have been collected in routine diagnostics over a period of six years (Schröttner et al., 2017; Kopf et al., 2021). These isolates were recovered exclusively from diagnostic cultures analyzed at the Institute for Medical Microbiology and Virology, University Hospital Carl Gustav Carus (Dresden, Germany). Whole-genome sequences were submitted to NCBI GenBank under Acc. Nos JAGIBR000000000-JAGICE000000000, applying the NCBI Prokaryotic Annotation Pipeline PGAP (Tatusova et al., 2016) as previously reported (Kopf et al., 2021). In addition, the remaining publicly available *W. chitiniclastica* genomes at NCBI as of April 2021 (n = 12) were included in this study. The corresponding datasets were retrieved in preassembled nucleotide FASTA files and GenBank files. These include the type strain DSM 18708^T^ (AQXD01000000) (Tóth et al., 2008), BM-Y (LVXD00000000) (Zhou et al., 2016), Strain 20 (LWST01000000) (Matos et al., 2019), SH04 (AOBV01000000) (Cao et al., 2013), and 8 genomes, which were submitted by the Centers for Disease Control and Prevention of the United States but are not associated to a citable publication as far as we know. The latter comprise of ATCC 51249 (NEFL01000000), F6512 (NEFK01000000), F6513 (NEFJ01000000), F6514 (NEFI01000000), F6515 (NEFH01000000), F6516 (NEFC01000000), F9188 (NEFE01000000), and G9145 (NEFC01000000). Isolation source, host, year of isolation, and geographical origins of isolates were taken from published research papers, otherwise estimated using dates/locations on public databases as indicated. Results are displayed in **Table 1**; **Supplementary Table S1**.

**Table 1:** Overview of the general genome features of the *W. chitiniclastica* genomes analyzed in this study.

Strain	Host	Isolation source	Location	Genome size (bp)	CDSs	rRNA	tRNA	CRISPR	Spacer	Phages	Acr/Aca
DSM 100374	Homo sapiens	Wound swab	Dresden, Germany	2079313	1961	3	53	2	79	4	4
DSM 100375	Homo sapiens	Wound swab	Dresden, Germany	2103638	1932	3	53	1	8	1	7
DSM 100676	Homo sapiens	Wound swab	Dresden, Germany	2139975	1953	3	51	2	188	3	8
DSM 100917	Homo sapiens	Wound swab	Dresden, Germany	2144768	1955	3	49	2	188	4	8
DSM 105708	Homo sapiens	Wound swab	Dresden, Germany	2084087	1969	3	52	2	15	4	9
DSM 105712	Homo sapiens	Wound swab	Dresden, Germany	2133608	1960	3	49	3	67	3	2
DSM 105838	Homo sapiens	Wound swab	Dresden, Germany	2069521	1910	3	54	3	69	5	9
DSM 105839	Homo sapiens	Wound swab	Dresden, Germany	2123437	1966	3	54	2	41	3	8
DSM 105984	Homo sapiens	Wound swab	Dresden, Germany	2120278	1965	3	49	3	78	3	8
DSM 106597	Homo sapiens	Wound swab	Dresden, Germany	2131555	1966	3	50	3	78	3	8
DSM 108045	Homo sapiens	Wound swab	Dresden, Germany	2090370	1950	3	53	2	38	1	10
DSM 108048	Homo sapiens	Wound swab	Dresden, Germany	2074016	1952	3	54	4	130	2	8
DSM 110179	Homo sapiens	Wound swab	Dresden, Germany	2119644	1965	3	49	3	78	3	8
DSM 110473	Homo sapiens	Wound swab	Dresden, Germany	2126147	1970	3	54	2	53	3	7
DSM 18708^T^	Wohlfahrtia magnitica	3rd stage larvae of fly	Mezöfalva, Hungary	1991020	1849	4	45	3	42	1	2
SH04	Chrysomya megacephala	-	Pudong, China	2181980	2132	12	56	3	106	7	10
BM-Y	Zebra	Pancreas	Shenzhen, China	2180519	2029	9	51	2	244	3	11
Strain 20	Chicken	Chicken carcass	Rio de Janeiro, Brazil	2123239	1958	3	48	3	59	1	2
ATCC 51249	Homo sapiens	Arm	New York, USA	2136105	1973	7	48	2	151	2	10
F6512	Homo sapiens	Foot	New York, USA	2120698	1968	7	52	2	40	1	6
F6513	Homo sapiens	Leg	New York, USA	2115422	1975	5	49	2	73	3	6
F6514	Homo sapiens	Oral lesion	New York, USA	2112239	1974	5	49	2	73	3	6
F6515	Homo sapiens	Ankle	New York, USA	2134718	2011	5	50	2	53	6	6
F6516	Homo sapiens	Arm	New York, USA	2071321	1892	7	48	2	39	2	9
F9188	Homo sapiens	Leg wound	Indiana, USA	2127263	1987	7	49	2	106	2	9
G9145	Homo sapiens	Wound	Colorado, USA	2182988	2017	5	51	2	107	2	10

### *In Silico* Genome Analysis

Functional genome analysis was performed using the freely available computational tools with default parameters from January till June 2021, unless indicated otherwise. Preassembled FASTA files were annotated using Prokka (Galaxy version 1.14.6+galaxy0) (Seemann, 2014) and strarmar (Galaxy Version 0.7.2+galaxy0) (Petkau, 2018). Results are displayed in **Figure 3**; **Supplementary Tables S6, S7**. Search for antimicrobial resistance profiling was extended by using the comprehensive antibiotic resistance database CARD (https://card.mcmaster.ca/) (Alcock et al., 2020) retaining “Perfect hit and Strict hit only” and “High-quality/coverage”. Results are displayed in **Figure 3**; **Supplementary Table S8**. Phage analysis was performed using PHASTER (PHAge Search Tool Enhanced Release) (https://phaster.ca/) (Arndt et al., 2016). Results are displayed in **Table 1**; **Supplementary Table S2**. Analysis of CRISPR (clustered regularly interspaced short palindromic repeats) and their associated (Cas) proteins was done using the CRISPRCasFinder (Couvin et al., 2018) (https://crisprcas.i2bc.paris-saclay.fr/). Only results with evidence levels 3 and 4 were included in the analysis. Results are displayed in **Table 1**; **Supplementary Table S1**. AcrFinder (http://bcb.unl.edu/AcrFinder/index.php) (Yi et al., 2020) was used for the detection of Anti-CRISPR (Acr) proteins. Results are displayed in **Table 1**; **Supplementary Table S3**.

### Phylogenetic Identification

For phylogenomic identification; genomic contigs were submitted to the Type Strain Genome Server at https://tygs.dsmz.de/ (Meier-Kolthoff and Göker, 2019). Gene sequence of the 16S rRNA gene and *rpoB* gene were retrieved from the previous results using Prokka (Galaxy version 1.14.6+galaxy0), and BLAST analysis for the homology of the 16S rRNA gene and the *rpoB* gene for the identification of *W. chitiniclastica* was performed on https://blast.ncbi.nlm.nih.gov (Altschul et al., 1990).

### Pan-Genome Assembly and Visualization

**Figure 1:**
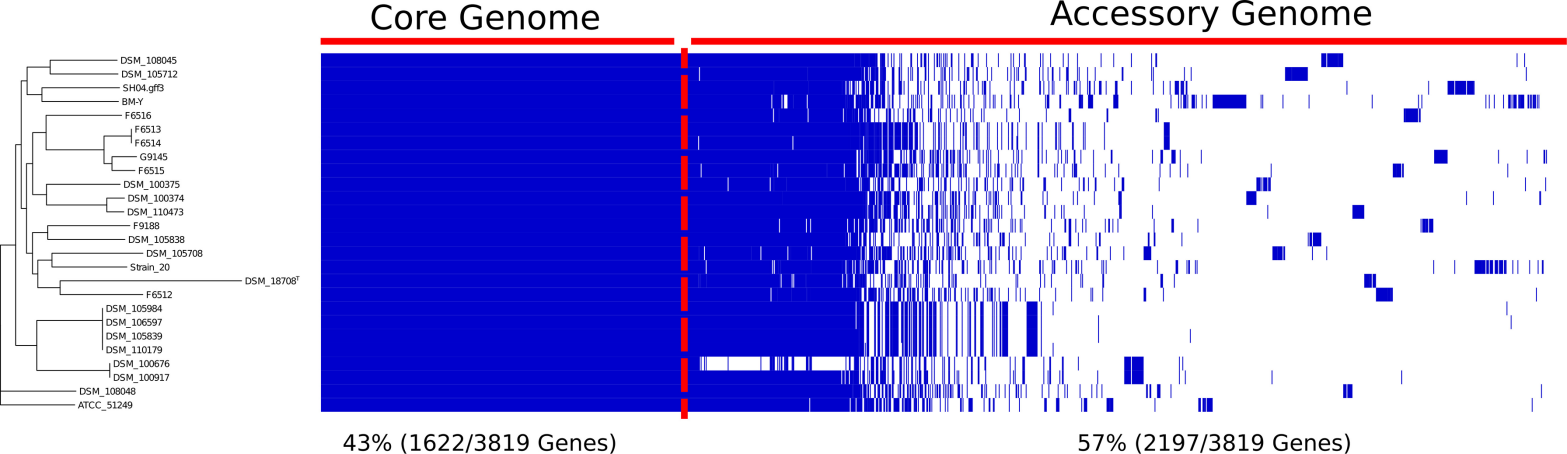
Linearized visualization of W. chitiniclastica pan-genome along with the phylogenetic tree. The pangenome was visualized based on the software Phandango (Hadfield et al., 2018).

Preassembled GenBank files were converted to GFF3 using the ‘Genbank to GFF3’ converter (Galaxy Version 1.1) (Stajich et al., 2002). Annotated GFF3 files of 26 *W. chitiniclastica* genomes were submitted to Roary (Galaxy Version 3.13.0+galaxy1) (Page et al., 2015) for pan genome analysis using default parameters. A gene-absence-presence data matrix was derived and visualized with Phandango (Hadfield et al., 2018). Results are displayed in **Figure 1**; **Supplementary Tables S4, S5**.

## Results

### Characterization of Central Genome Features

Over a period of six years, a total of 14 *W. chitiniclastica* strains were recovered from wound swabs in routine diagnostics (**Table 1**) (Kopf et al., 2021). The genome size of these *W. chitiniclastica* isolates, linked in terms of geographic location and isolation source, ranged from 2.07 to 2.14 million bases with an average of 1941 predicted genes (**Table 1**). To broaden the picture, we extended our study to include all *W. chitiniclastica* genomes, which were publicly available at NCBI as of April 2021. Thus, we arrived at 26 genomes comprising 22 isolates from human sources and 4 strains from animal sources (**Table 1**). Genome size ranged from 1.99 to 2.18 million bases with an average of 1967 predicted genes, 51 tRNAs, and five rRNAs. Notably, all isolates from Dresden and Strain 20 have three rRNAs, whereas the other genomes contain an average of seven rRNAs. Interestingly, BM-Y (n = 4) and Strain 20 (n = 2) contain multiple copies of the 16S rRNA gene (**Supplementary Table S1**).

The CRISPRCasFinder (Couvin et al., 2018) was used for the identification of potential genes encoding for CRISPR (clustered regularly interspaced short palindromic repeats) arrays and their associated (Cas) proteins. Because CRISPR arrays of evidence levels 1 and 2 are potentially invalid (Couvin et al., 2018), we focused on the results of evidence levels 3 and 4, which are considered as highly likely candidates. All strains contain CRISPR repeats, spacers, and the cas cluster CAS-Type IF (**Table 1**; **Supplementary Table S1**). On average, the isolates contain two CRISPR and 85 spacers. DSM 108048 stands out with four CRISPR and 130 spacers, while DSM 100375 contains only one potential CRISPR sequence and eight spacers. The highest number of spacers was detected in Strain 20 isolated from an animal source. Interestingly, all *W. chitiniclastica* isolates also contain genomically encoded anti-CRISPR (*acr*) genes (**Table 1**). DSM 108048, SH04, ATCC 51249 and G9145 stand out with 10 genomically encoded Acr proteins, whereas the type strain DSM 18708, Strain 20, and DSM 105712 contain only two sequences. Although some *acr* genes were labeled ‘low confidence’ (**Supplementary Table S3**), the majority are homologs of known Acr proteins, making the actual presence of potential anti-CRISPR proteins highly likely.

### Phylogenetic Identification

**Figure 2:**
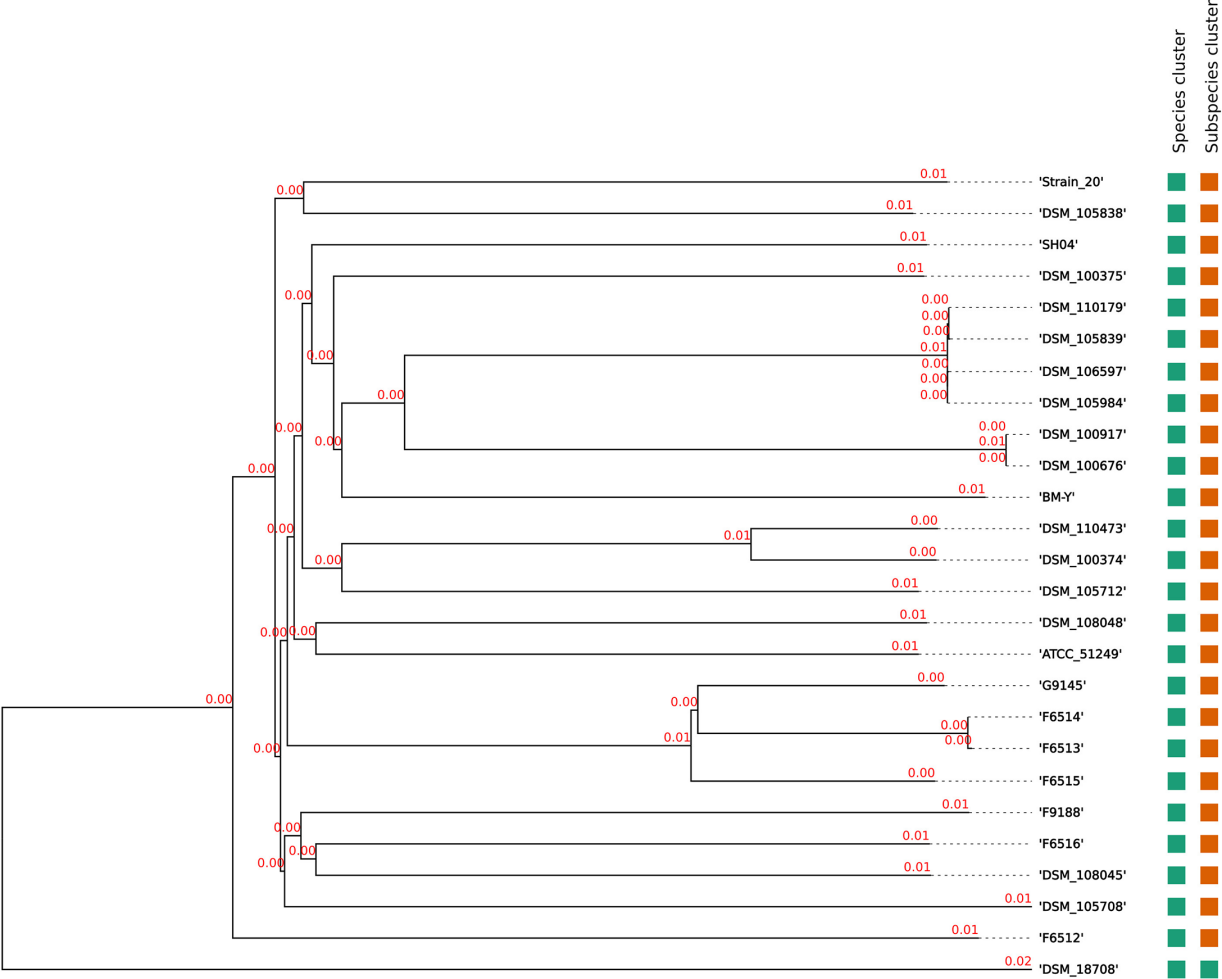
Phylogenomic tree of the *W. chitiniclastica* species and subspecies delineation based on the GBDP phylogenetic analyses retrieved and modified based on the Type (Strain) Genome Server (TYGS). The tree was inferred with FastME 2.1.6.1 (Lefort et al., 2015) from GBDP distances calculated from genome sequences and was subjected to a clustering using established thresholds for delineating species (DDH > 70%) (Meier-Kolthoff et al., 2013) as well as subspecies (DDH > 79%) (Meier-Kolthoff et al., 2014). The branch lengths are scaled in terms of GBDP distance formula d5 and are represented in red numbers. The numbers above branches are GBDP pseudo-bootstrap support values >60% from 100 replications, with an average branch support of 83.0%.

Genome-based-taxonomy analysis of all strains revealed correct assignment to *W. chitiniclastica.* Hereby, digital DNA:DNA hybridization (dDDH) values of 74.0-75.2% were computed against the type strain DSM 18708^T^ and therefore fulfilling the criteria for correct bacterial species identification (Meier-Kolthoff and Göker, 2019) (**Supplementary Table S1**). Construction of a phylogenomic tree based on whole-genome sequences revealed that all strains cluster in one subclade with the type strain DSM 18708^T^, and 25 strains form a subspecies using a 79% dDDH threshold (Meier-Kolthoff et al., 2014) (**Figure 2**). Surprisingly, the type strain does not belong to the subspecies. Furthermore, 16S rRNA gene and *rpoB* gene sequences were compared for sequence similarity to other sequences using the BLAST database. All isolates were identified with a minimum of 98% out of 100% sequence identity to corresponding 16S rRNA and *rpoB* reference genes, respectively, leading to the correct assignment of *W. chitiniclastica* (**Supplementary Table S1**). Notably, the additional 16S rRNA gene copies of BM-Y (n = 4) and Strain 20 (n = 2) revealed correct taxonomic assignment and share high homology, ranging from 99.7%-99.9%.

### Pan-Genome Construction

We constructed the pan-genome of *W. chitiniclastica* encompassing 26 genomes, which represents the first and largest analysis of this type to date. Roary (Page et al., 2015) was used to cluster the genes encoding complete protein sequences into core (hard core and soft core) and accessory (shell and cloud) genomes. The core genome is shared by every genome tested in this study and its genes are often related to housekeeping functions. It can further be divided into hard core genes, which are defined to be present in >99% of the genomes, and soft core genes, which are present in 95–99%. The accessory genome is shared by a subset of the genomes and is associated with, but not limited to, pathogenicity or environmental adaptation. It is subdivided into shell genes, which are present in 15–95%, and cloud genes, which are found in less than 15% of genomes. The latter include singletons or genes found in only one of the genomes.

The pan-genome of all 26 *W. chitiniclastica* isolates comprises 3819 genes; 1622 core genes (43%), and 2197 accessory genes (57%) (**Figure 1**) with 1117 unique genes (29%) defined as genes only present in one strain. We also observed a remarkable abundance of 1240 (32%) genes with an unknown function. The core genome divides into 1175 hard (31%) and 447 soft core (12%) genes. Notably, 92% of the core genes (1494/1622) encode a known function, while only 8% (128/1622) are assigned to hypothetical proteins. The accessory genome comprises 635 shell (17%) and 1562 cloud (41%) genes. Remarkably, 51% (1122/2197) of the accessory genes code for hypothetical proteins, a fact that highlights the limited characterization of the *W. chitiniclastica* genome.

The majority of core genes encodes for protein families associated with housekeeping functions such as amino acid metabolism, energy production and translation, to name but a few (**Supplementary Table S4**). Genes associated with defense mechanisms such as antimicrobial resistance (AMR) genes are mainly present in the accessory genome (**Supplementary Table S5**). Notably, major multidrug efflux systems, on the other hand, are encoded within the core genome (**Supplementary Table S4**). These include the adenosine triphosphate (ATP)-binding cassette (ABC) superfamily, resistance nodulation-division (RND) family, major facilitator superfamily (MFS), small multidrug resistance (SMR) family, multidrug and toxic compound extrusion (MATE) family, proteobacterial antimicrobial compound efflux (PACE) family, and *p*-aminobenzoyl-glutamate transporter (AbgT) family. In addition, we detected several protein families encoding TRAP transporters and TonB dependent transport systems.

The generated phylogenetic tree of the pan-genome shows three main clades (**Figure 1**). DSM 108048 and ATCC 51249 are clustered in a single lineage (clade 1), and clade 2 is composed of six isolates from Dresden. The remaining isolates are grouped in clade 3, suggesting potential spread and transmission between hosts without any clear geographical links or host association.

### Prediction of Arsenic Resistance

**Figure 3:**
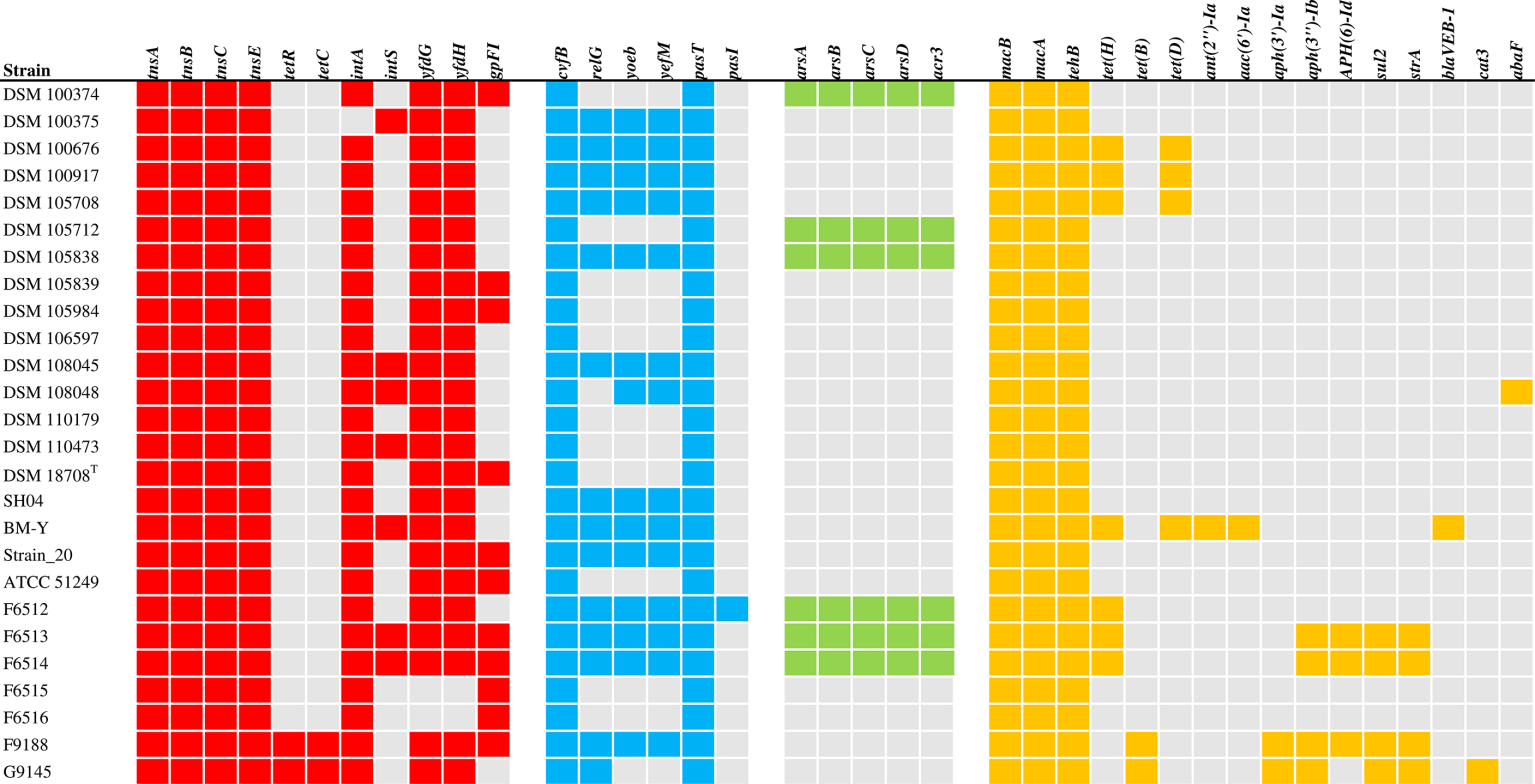
Heat-map visualizing genes derived from mobile genetic elements (red), and genes associated with putative virulence traits (blue), arsenic resistance (green) and antimicrobial resistance (yellow). Cell colors indicate the presence of genes: grey (absence); red, blue, green and yellow (presence).

Pan-genome analysis revealed two protein families encoding for arsenic resistance proteins within the core genome (**Supplementary Table S4**). Interestingly, genes belonging to the ubiquitous *ars* operon (*arsRDABC*) such as *arsC*, *arsD, arsA*, and *arsB* (Carlin et al., 1995) were only detected in DSM 100375, DSM 105712, DSM 105838, F6512, F6513, and F6514 (**Figure 3**). In addition, these six isolates contain the gene for the inorganic arsenic efflux pump *acr3* (Fekih et al., 2018).

### Prediction of Bacteriophages and Other Mobile Genetic Elements

Prediction of prophage sequences within the *W. chitiniclastica* genomes revealed a total of 75 prophages (**Table 1**), 18 of which are classified as intact (**Supplementary Table S2**). The latter are most likely to be complete and functional, and were found in 16 different genomes all isolated from a human source. The most common intact phage was identified either as Mannheimia (n = 12) or Enterobacteria phage (n = 4) (**Supplementary Table S2**). The remaining prophages were classified as “questionable” (n = 4) and “incomplete” (n = 53). Notably, none of the strains isolated from an animal source contained intact phages.

We then extended our search for the presence of other mobile genetic elements as it can improve the fitness and enables bacteria to acquire new AMR genes (Kottara et al., 2018; Stalder et al., 2019). Each genome contained genes homologous to Tn*7* transposon genes such as *tnsA, tnsB, tnsC*, and *tnsE* (Peters, 2014; Babakhani and Oloomi, 2018) (**Figure 3**). This is consistent with pan-genome analysis, which revealed a ubiquitous presence of a transposase encoding protein family within the core genome (**Supplementary Table S4**). Interestingly, the accessory genome showed numerous additional transposases as well as integrases and phage proteins (**Supplementary Table S5**). Detailed genome analysis based on Prokka confirmed these results (**Supplementary Table S6**). For example, F9188 and G9145 harbor Tn*10* encoded tetracycline resistance genes *tetR* (Smith and Bertrand, 1988) and *tetC* (Babakhani and Oloomi, 2018) (**Figure 3**). Prophage integrase *intA* gene, which is necessary for the integration of the phage into the host genome by site-specific recombination (Kirby et al., 1994), was present in all genomes except DSM 100375. Integrase gene *intS* (Panis et al., 2010), on the other hand, was only detected in seven genomes including DSM 100375. Notably, BM-Y contains five *intS* copies (**Supplementary Table S6**). Additional genome-integrated phage genes such as *yfdh, yfdg* (Rudd, 1999) and *gpFI* (Popovic et al., 2012) were distributed among the *W. chitiniclastica* genomes (**Figure 3**).

### *In Silico* Annotation of Potential Virulence Traits

We analyzed all genomes including the type strain DSM 18708^T^ with respect to potential virulence traits (**Supplementary Table S6**). To begin with, all isolates harbor the conserved virulence factor B (*cvfB*) (Matsumoto et al., 2007) and genes encoding for active multidrug efflux systems. The latter are one of the major mechanisms of bacterial resistance to drugs. For example, we detected the multidrug resistance transporter gene *mdtA* (Nagakubo et al., 2002), the MATE transporter genes *norM* (Nishino et al., 2006) and *mepA* (Kaatz et al., 2006), the TonB dependent transport genes *tdhA* (Thomas et al., 1998)*, exbD*, and *exbB* (Bosch et al., 2002; Holden et al., 2012), and multidrug efflux pumps genes *acrB* and *arcA* (Eicher et al., 2009), to name but a few. Secretion systems are another key element in the pathogenesis of bacterial infections, and all genomes harbor copies of the type II secretion (TS2) genes *xcpT* and *epsF* (Sandkvist, 2001).

Other meaningful elements involved in pathogenicity are toxins. Although *W. chitiniclastica* has not been reported to be a prolific toxin-producing organism, all genomes were manually searched for potential candidates. Based on our *in silico* results, 14 isolates contain the toxin *relG* gene (Korch et al., 2015). Notably, F9188, G9145 and SH04 harbor two copies of *relG*.

Toxin-antitoxin (TA) modules are ubiquitous among bacteria, and are involved in diverse physiological processes including virulence (Lobato-Márquez et al., 2016). Interestingly, the core genome harbors a protein family coding for the type II toxin-antitoxin system RatA (**Supplementary Table S4**), which is described as antisense RNA that blocks the accumulation of the mRNA for the TxpA toxin (Silvaggi et al., 2005). However, the corresponding *txpA* gene appears to be missing. Complete TA modules, on the contrary, can be found in the accessory genome (**Supplementary Table S5**). These include the RelE/ParE family, which encompasses several smaller toxin families including YoeB (Anantharaman and Aravind, 2003). In-depth genome analysis using Prokka (Seemann, 2014) confirmed these results by revealing genes encoding for the TA type II system YefM-YoeB (Norton and Mulvey, 2012) in 14 *W. chitiniclastica* strains (**Figure 3**). Notably, SH04 harbors two copies of *yefM* and *yoeB*. The PasTI is another known TA module (Norton and Mulvey, 2012), and F6512 contains both genes, *pasT* and *pasI*, respectively. The remaining 25 isolates only harbor the *pasT* gene.

### *In Silico* Analysis of Antimicrobial Resistance Genes

In order to perform an thorough and comprehensive search for AMR genes, we extended our previous analysis using the comprehensive antibiotic resistance database CARD (Alcock et al., 2020) retaining ‘Perfect’ and ‘Strict’ hits only while excluding ‘Loose’ hits (**Supplementary Table S8**). Noteworthy, the ‘Strict’ algorithm represents a flexible sequence variation but lies within the curated BLAST bit score cut-off (Alcock et al., 2020), and by that, making a correct identification highly feasible. The ‘Loose’ algorithm, on the other hand, works outside of the detection model cut-off to provide detection of new and more distant homologs of AMR genes. Although it could help to identify potential resistance genes and/or shed light on new unknown modifications, *in silico* results based on loose hits should always be taken with caution and require further research. In order to gain high-quality results we decided to restrict the CARD search to ‘Perfect’ and ‘Strict’ hits, and combine the outcome with results gained by Prokka (Seemann, 2014) and strarmar (Petkau, 2018). Summarized results are displayed in **Figure 3**, and detailed information can be retrieved from **Supplementary Tables S6, S7**.

Genes coding for macrolide-specific efflux pumps (*macA* and *macB*) (Kobayashi et al., 2000; Yum et al., 2009), and tellurite resistance methyltransferase (*tehB*) (Choudhury et al., 2011) were detected in all 26 genomes. Tetracycline resistant efflux protein *tetH* gene (Roberts, 2005) was found in 7 genomes; notably, BM-Y harbors double *tetH* genes. In addition, DSM 100676, DSM 100917, DSM 105708, and BM-Y contained the tetracycline repressor protein *tetD* gene (Roberts, 2005). G9145 and F9188, on the other hand, harbor the tetracycline efflux protein *tetB* gene, which confers resistance to tetracycline, doxycycline, and minocycline, but not tigecycline (Roberts, 2005). BM-Y contains two aminoglycoside resistance genes encoding for the adenylyltransferase *ant(2’’)-Ia* (Cox et al., 2015) and acetyltransferase *aac(6’)-Ia* (Parent and Roy, 1992). The remaining aminoglycoside resistance genes such as *aph(3’’)-Ib*, *aph(3’)-Ia* (Tauch et al., 2000), and *aph-ld* (Cao et al., 2013)*-Id* (Chiou and Jones, 1995) were detected in F6513, F6515, G9188, and F9188, respectively. Sulfonamide resistance gene *sul2* and streptomyicn resistance gene *strA*, which have been described to be encoded within the same resistance gene cassette (Anantham and Hall, 2012), were detected in F6513, F6514, G9145, and F9188. In addition, BM-Y harbored a gene encoding for the VEB-1 beta-lactamase (Nordmann and Naas, 1994), and G9145 contains the chloramphenicol acetyltransferase *cat3* gene (Vassort-Bruneau et al., 1996). Finally, yet importantly, DSM 108045 contains the antibiotic efflux pump encoding *abaF* gene, which has been reported, when expressed, to increase fosfomycin resistance (Sharma et al., 2017).

## Discussion

In the past 12 years, several case reports have shown that *W. chitiniclastica* is capable of causing sepsis and bacteremia in humans labeling this organism as a newly underestimated pathogen. However, little is known about its pathogenicity and genome content. Our current study, analyzing all publicly available *W. chitiniclastica* genomes to date, highlights significant genomic characteristics including potential virulence factors, and AMR genes. Moreover, we provide the first pan genome analysis and shed light on the core features of *W. chitiniclastica.*

### Distinct Genomic Characteristics of *W. Chitiniclastica* and Their Effect on the Assessment of Microbial Diversity

Genome size ranged from 1.99 to 2.18 million bases with an average of 1967 predicted genes, 51 tRNAs, and 5 rRNAs. Interestingly, all isolates from Dresden and Strain 20 harbor 3 rRNAs, while the remaining genomes contained an average of 7 rRNAs. It has recently been proposed that multiple rRNA operons confer a selective advantage to respond quickly and grow rapidly in environments characterized by fluctuations in resource availability (Stevenson and Schmidt, 2004). Based on this hypothesis, with 12 and 9 rRNAs, respectively, SH04 and BM-Y should have a fitness advantage when compared to the other isolates. In addition, BM-Y and Strain 20 harbor multiple 16S rRNA gene copies; a fact that has not been reported for any *W. chitiniclastica* strain yet. Although most bacterial genomes exhibit only one or two 16S rRNA genes (Armougom, 2009), some microbes contain multiple or varying numbers (Rainey et al., 1996; Acinas et al., 2004). For example, *Bacillus subtilis* has 10 copies (Stewart et al., 1982) and *Clostridium paradoxum* has up to 15 copies with heterogeneous intervening sequences (Rainey et al., 1996). Those multiple copies are often associated with nucleotide sequence variability (Rainey et al., 1996; Acinas et al., 2004), and/or provide insufficient taxonomic resolution power at the species level or with closely related species (Janda and Abbott, 2007). Our study indicates that the phylogenetic identification of those additional 16S rRNA gene copies of BM-Y and Strain 20 provides correct classification; however it can lead to an overestimation in terms of abundance and diversity composition (Armougom, 2009), a fact that one has to keep in mind within the scope of a thorough microbial community profiling. 16S rRNA-based identification generally faces a number of challenges, including sequencing error (Schirmer et al., 2015), primer bias (Klindworth et al., 2012), and varying discrimination power between variable regions (Sune et al., 2020) and certain bacterial genera (Roux et al., 2011). In light of these drawbacks, an alternative, or at least complementary taxonomic markers, should be considered. Our study indicates that both dDDH and *rpoB* based analysis have proven a worthy identification method for *W. chitiniclastica*. While dDDH is a very costly and time-consuming technique, the *rpoB* gene has emerged as a new marker gene candidate for phylogenetic analyses and identification of bacteria (Adékambi et al., 2009). Although the *rpoB* gene does not have a database currently as comprehensive as that of the 16S rRNA gene (Bondoso et al., 2013), the approach has a number of advantages. For example, *rpoB* excels with an increased phylogenetic resolution on the genus level or lower, and it is a single-copy protein-encoding gene enabling a phylogenetic analysis on amino acid and nucleotide level (Case et al., 2007; Adékambi et al., 2009). However, since neither the dDDH nor the *rpoB* approach is widely established in clinical routine diagnostics yet, 16S rRNA-based identification most likely remains by far the most frequently used method. Nevertheless, in case of doubtful results, additional methods should be considered as an alternative or complement.

### Mobile Genetic Elements and Their Putative Effect in Shaping the Genetic Diversity of *W. Chitiniclastica*

Genome scanning for phages and MGE revealed the ubiquitous presence of transposons and bacteriophages within all *W. chitiniclastica* isolates tested in this study, and we believe that they are some of the key elements in shaping the genetic diversity within the *W. chitiniclastica* clade. Interestingly, all genomes harbor CRISPR-Cas elements, which are described to constitute the adaptive immune system in prokaryotes in order to provide resistance against invasive genetic elements including viruses, plasmids, and transposons (Barrangou et al., 2007). Therefore, in theory, *W. chitiniclastica* should be well equipped against its invasion. However, the ubiquitous distribution of MGEs among prokaryotes suggests that CRISPR systems are not always functional (Grissa et al., 2007), participate in other processes, such as signal transduction and gene regulation (Westra et al., 2014; Faure et al., 2019), and/or have a yet undiscovered function (Aydin et al., 2017). Moreover, many phages have evolved an anti-CRISPR system that inhibits the CRISPR immune response of their host (Landsberger et al., 2018). Although Acr proteins were first discovered in *Pseudomonas* phages and other prophages (Bondy-Denomy et al., 2013), they have also been detected in other prokaryotes such as *Moraxella bovoculi* (Marino et al., 2018). In fact, >30% of *P. aeruginosa* strains carrying a CRISPR-Cas system also encode one or more cognate *acr* genes (van Belkum et al., 2015); therefore, the ubiquitous presence of genome-encoded Acr proteins among *W. chitiniclastica* may partly explain the widespread distribution of MGE and phage-related genes. Although the conditions and extent to which these immunosuppressive genes allow bacteriophages to persist in their bacterial host remain unclear (Landsberger et al., 2018), the balance between CRISPR-Cas immunity and Acr activities may be a central element in shaping the genetic diversity of *W. chitiniclastica* including antimicrobial resistome expansion.

### Pan-Genome Composition of *W. Chitiniclastica*

To begin with, the pan-genomic phylogenetic tree indicated no clear host or geographical clustering suggesting a potential spread and transmission. Although six strains isolated from Dresden clustered within a subclade, the analysis might be limited in terms of capturing total diversity due to the fact that the majority are from the same location, and only four are associated with an animal source. With hopefully increasing numbers of available genomes from various locations, we recommend repeating the phylogenetic pan-genome analysis to see whether a certain niche specificity emerges.

The composition of the pan-genome revealed a core genome of 43%, which appears to be conserved when compared to other reported species such as *Clostridium perfringens* (12.6%) (Kiu et al., 2017), *Aliarcobacter butzleri (22%)* (Buzzanca et al., 2021)*, Staphylococcus aureus* (32%), *Pseudomonas aeruginosa (26%)* (Costa et al., 2020), *Klebsiella pneumoniae* (26%) and *Salmonella enterica* (16%) (McInerney et al., 2017), to name but a few. Microbes with large and diverse accessory genomes, on the other hand, are considered metabolically versatile species with the ability to migrate to new niches, and to adapt to changing environmental conditions (Rouli et al., 2015; Costa et al., 2020). Accessory genes are often acquired by Horizontal gene transfer (HGT) (Costa et al., 2020), and are related to virulence, antimicrobial defense or confer a fitness advantage (Jordan et al., 2001). Therefore, species with a large and conserved core genome often lack a diverse pool of virulence and AMR factors. This is congruent with recent studies regarding *Bordetella pertussis*. Members of this species have a large core genome (59%) suggesting that due to this low genomic diversity, antibiotics and vaccines are quite effective against this species (Tettelin et al., 2008; Carbonetti, 2016; Costa et al., 2020). This is consistent with recent observations regarding *W. chitiniclastica* isolates, which are described to be susceptible to the majority of known antibiotics with the exception of fosfomycin (Schröttner et al., 2017; Matos et al., 2019), and by that, underlines the assumption that members of this species appear to be metabolically conserved when compared to others. However, as mentioned above, with increasing numbers of available genomes a large-scale pan-genome analysis is recommended to confirm and/or reevaluate the findings.

### Further Evidence of a Previously Newly Described Subspecies of *W. Chitiniclastica*

Originally isolated from *Wohlfahrtia magnifica* larvae in Hungary (Tóth et al., 2008), there is increasing evidence that *W. chitiniclastica* inhabits diverse niches and environmental habitats such as soil (Sanyal et al., 2016), humans (Schröttner et al., 2017), other mammals (Thaiwong et al., 2014; Diaz-Delgado et al., 2015), fish (Reddy and Mastan, 2013), and diverse flies (Lysaght et al., 2018; Maleki-Ravasan et al., 2020). In most of these published studies, identification was based on 16S rRNA gene sequence, which is known to lack sufficient resolution to distinguish between closely related species (Roux et al., 2011). The dDDH analysis performed during this work surprised with further evidence of a previously newly described subspecies of *W. chitiniclastica* (Kopf et al., 2021) (**Figure 2**). Originally thought to be the adaptation to a human environment and geographic location (Kopf et al., 2021), this work rather suggest a broad host and environmental range of *W. chitiniclastica*. This observation is also reflected in the pangenomic phylogenetic tree (**Figure 1**). The fact that *W. chitiniclastica* can colonize different species should be considered an advantage for the bacterium. However, on the other hand, it also poses an increased risk for zoonotic transmission, whose dynamic interactions between humans, animals, and pathogens should be considered in the context of the “One Health” approach (Welch et al., 2007; Cantas and Suer, 2014).

### Prediction of Arsenic Resistance

*W. chitiniclastica* has recently been detected in arsenic-affected soils from Bangladesh (Sanyal et al., 2016) indicating its ability to inhabit different habitats including soil, humans, and animals (Tóth et al., 2008; Thaiwong et al., 2014; Diaz-Delgado et al., 2015; Sanyal et al., 2016; Schröttner et al., 2017; Matos et al., 2019). Arsenic occurs naturally in aquatic and terrestrial environments, and despite its relatively low abundance, the high toxicity of arsenic derivatives is considered a severe problem of public health worldwide (Fekih et al., 2018). In this process, microorganisms are known to play a crucial role in global arsenic geocycles (Zhu et al., 2014) subsequently leading to the ubiquitous presence of arsenic resistance genes among microbes (Fekih et al., 2018). With the presence of arsenic-resistant protein families within the core genome, *W. chitiniclastica* is no exception. However, genes of the common *arsRDABC* operon were only detected in six genomes. Notably, the regulator protein-encoding *arsR* gene, which acts as a repressor of the *arsRDABC* operon in the absence of arsenic (Cai et al., 2009), appears to be missing. These findings indicate the development of a yet unknown regulation and/or arsenic tolerance mechanism distributed among *W. chitiniclastica*. Moreover, these 6 isolates harbor the arsenic efflux pump *acr3* gene (Fekih et al., 2018), which is surprising as most prokaryotic species are described to have either an *arsB* or *acr3* gene (Yang et al., 2012). Notably, arsenic-resistant bacterial isolates from highly arsenic-contaminated soils showed a predominance of *acr3* genes over *arsB* genes (Cai et al., 2009) suggesting that DSM 100375, DSM 105712, DSM 105838, F6512, F6513, and F6514 have a severe fitness advantage in highly enriched arsenic habitats.

### *In Silico* Profiling of Potential Virulence Traits

To detect possible genomic signatures linked to virulence, all genomes were manually searched for genes putatively associated with host-pathogen interaction. The ubiquitous presence of diverse multidrug efflux systems emphasizes a central role in the pathogenesis of *W. chitiniclastica*. For example, the core genome harbors a PACE efflux transporter, which is described to confer resistance to a wide range of biocides used as disinfectants and antiseptics, and are encoded by many Gram-negative human pathogens (Hassan et al., 2018). Other potential virulence components are represented by membrane-associated proteins, like TonB dependent transport systems, which are involved the in virulence of *Shigella dysenteriae, Haemophilus influenzae* and *E. coli*, to name but a few (Reeves et al., 2000; Torres et al., 2001; Morton et al., 2012). Moreover, the ubiquitous presence of the conserved virulence factor B (*cvfB*) suggests a central role in the virulence of *W. chitiniclastica*. Recent studies showed that deletion of CvfB results in reduced virulence in *S. aureus* and decreased production of hemolysin, DNase, and protease (Matsumoto et al., 2007), which further emphasized its importance for pathogenicity. However, in-depth research including target specific manipulations is required to unravel its function in *W. chitiniclastica.*

TA modules are involved in diverse physiological processes providing bacteria with pronounced fitness advantages dependent on toxin expression levels and the specific environmental niche occupied (Ma et al., 2021). These include bacterial adaptation to hostile environments, mediating stress response, stabilization of chromosomal regions, and bacterial survival during infection (Lobato-Márquez et al., 2016). Our *in silico* analysis indicates the presence of the TA system YefM-YoeB (Norton and Mulvey, 2012) in 14 *W. chitiniclastica* strains. This TA module has been described to be involved in the niche-specific colonization, stress resistance, and survival inside the host (Norton and Mulvey, 2012). Although the natural habitat of *W. chitiniclastica* is not well investigated, we could envision that TA systems might be involved in invading different habitats including persistence as part of a polymicrobial infection. Moreover, F6512 harbors a second TA system comprised of the toxin PasT and the antitoxin PasI. The PasTI module assures cell formation in the presence of antibiotics and increases pathogen resistance to nutrient limitation as well as oxidative and nitrosative stresses (Norton and Mulvey, 2012). Notably, the remaining 25 isolates only harbor the *pasT* gene, whose function has recently been reannotated based on new experimental evidence. While it was shown that PasT sustains antibiotic tolerance, and is critical for the formation or survival of ciprofloxacin-tolerant cells, the function of PasTI as a TA system could not be confirmed (Fino et al., 2020). Instead, the supposed toxin PasT is a bacterial homolog of mitochondrial protein Coq10 suggesting a central role in respiratory electron transport by acting as an important accessory factor in the ubiquinone-dependent electron transport chain (Fino et al., 2020). This leaves us to speculate, whether the *pasT* gene of *W. chitiniclastica* is primarily involved in virulence and/or energy production.

The secretion of proteins and toxins have a major role in the pathogenesis of bacterial infections, and several highly specialized pathways have evolved for this purpose such as the T2S system. The latter has been widely discovered in a number of bacterial species including several human pathogens like *Chlamydia trachomatis, Escherichia coli, Klebsiella pneumoniae, Legionella pneumophila, Vibrio cholerae*, and *P. aerunginosa*, to name but a few (Sandkvist, 2001; Durand et al., 2003; Cianciotto and White, 2017). In general, proteins secreted by T2S systems are associated with the destruction of various tissues, cell damage and diseases such as proteases, cellulases, pectinases, phospholipases, lipases, and toxins (Sandkvist, 2001). Based on our *in silico* analysis, *W. chitiniclastica* harbors, for example, a copy of the *xcpT* gene, which encodes for pseudopilin XcpT of the T2S machinery of *P. aeruginosa* (Sandkvist, 2001; Durand et al., 2003). Notably, assembly of the type II pseudopilus also confers increased bacterial adhesive capabilities (Durand et al., 2003), which raises the question, whether its function in *W. chitiniclastica* is primarily involved in adhesion and/or endotoxin secretion. Unfortunately, the analysis regarding the toxin profile of *W. chitiniclastica* provided limited information. Although some isolates harbor the toxin-encoding gene *relG*, which is described to inhibit mycobacterial growth when expressed independently (Korch et al., 2015), other exotoxin encoding genes appear to be missing or are yet unknown. Based on this observation, *W. chitiniclastica* seems to contain a limited toxin profile when compared to other prolific toxin-producing organisms like *C. perfringens* (Kiu et al., 2017), making the participation of XcpT in bacterial cell adhesion feasible. However, T2S modules are not restricted to exotoxin secretion; in fact, they can export a wide range of substances. For example, in *V. cholerae* the T2S system supports the secretion of cholera toxin, hemagglutinin-protease, and chitinase (Connell et al., 1998; Davis et al., 2000; Sandkvist, 2001). Notably, *W. chitiniclastica* is known to have strong chitinase activity (Tóth et al., 2008) suggesting that T2S systems might be involved in its secretion.

### *In Silico* Analysis of Antimicrobial Resistance Genes

Previous studies have reported *W. chitiniclastica* to be susceptible to the majority of known antibiotics with the exception of fosfomycin (Schröttner et al., 2017; Matos et al., 2019; Kopf et al., 2021). This is in line with our *in silico* analysis, which showed that the majority lacks essential AMR genes suggesting a broad susceptibility against several clinical important antibiotics including β-lactamases and fluoroquinolones. This is congruent with recent case reports, where infections caused by *W. chitiniclastica* were successfully treated with levofloxacin (Schröttner et al., 2017; Bueide et al., 2021) and cephalosporins (Rebaudet et al., 2009; Campisi et al., 2015; Suryalatha et al., 2015; Snyder et al., 2020; Bueide et al., 2021), respectively. However, it should be noted that BM-Y carries a *bla*_VEB-1_ gene cassette, thus conferring resistance to ceftazidime and ampicillin as previously reported (Zhou et al., 2016).

Surprisingly, the previously reported fosfomycin resistance (Schröttner et al., 2017; Matos et al., 2019; Kopf et al., 2021) is not reflected within the core resistome profile. DSM 108045 harbors the MFS transporter gene *abaF*, which is described to confer resistance to fosfomycin (Sharma et al., 2017), but apart from several hits for multidrug efflux proteins within the core genome, we did not detect any known fosfomycin resistance genes such *fosA, fosC*, or *fomB* (Silver, 2017), to name but a few. Our results rather indicate a natural fosfomycin resistance most likely based on a yet unknown resistance mechanism as previously anticipated (Kopf et al., 2021). Macrolide resistance genes *macA* and *macB*, on the other hand, are encoded in the core genome. Unfortunately, there are no case studies available, that either support or deny our observation. Nevertheless, based on our *in silico* analysis macrolide resistance appears to be feasible, although further research is still required to uncover the macrolide resistance profile fully. Moreover, *W. chitiniclastica* appears to be resistant to tellurite, which is not surprising, since potassium tellurite was used intensively as an antimicrobial agent in the past, and as a consequence, many Gram-positive and Gram-negative bacteria developed resistance (Valková et al., 2007). Notably, tellurite resistance genes have also been reported to increase oxidative stress resistance in bacteria (Valková et al., 2007), which is another explanation for their core genome presence in *W. chitiniclastica*.

Additional genes involved in antimicrobial defense are distributed within the accessory genome indicating resistome expansion due to enormous selective pressure. For example, *in silico* analysis with respect to aminoglycosides revealed putative resistance in BM-Y, F6513, F6514, F9188 and G9145. Moreover, four isolates harbor sulfonamide resistance genes, while the remaining 22 strains appear to be susceptible. This is in line with recent case reports, in which *W. chitiniclastica* was susceptible to diverse antibiotics including trimethoprim/sulfamethoxazole (Chavez et al., 2017; Katanami et al., 2018; Connelly et al., 2019; Snyder et al., 2020; Bueide et al., 2021), while the first reported case in South Africa surprised with trimethoprim/sulfamethoxazole resistance (Hoffmann et al., 2016). A similar picture was observed for tetracycline resistance, which showed a rather diverse distribution among the isolates. This observation is also reflected in recent case and research studies, in which some isolates were susceptible to tetracyclines (Almuzara et al., 2011; Nogi et al., 2016), and some resistant (Snyder et al., 2020; Kopf et al., 2021). The presence of Tn*10* encoded tetracycline resistance genes *tetR* and *tetC* (Babakhani and Oloomi, 2018) further emphasizes the assumption that the majority of resistance genes within *W. chitinclastica* genomes were required *via* HGT. Notably, our data indicate that the type strain DSM 18708^T^ does not encode any additional clinical relevant AMR genes, while other strains harbor comparatively more. In particular BM-Y, F6513, F6514, F9188 and G9145 acquired an extended AMR profile; however, this is still limited when compared to other Gram-negative pathogens such as *Acinetobacter lwoffii* (Hu et al., 2011). Nevertheless, there is an increasing incidence of drug resistance within the *W. chitiniclastica* clade, whose development should be observed with caution.

## Conclusion

The present study provides novel insights on the genetic diversity and pan-genome composition of *W. chitiniclastica* a rare but potential new emerging human pathogen. Our analysis of all publicity available strains indicate a surprisingly conserved pan-genome without clear host or geographical clustering suggesting a potential spread and transmission. However, with an increasing number of available genomes, reanalysis is strongly recommended to confirm and/or reevaluate the findings. *In silico* genome studies revealed first insights into genomic features including putative virulence factors and AMR genes, that potentially influence pathogenicity. Interestingly, no clear toxin profile could be determined suggesting an alternative virulence profile. Our results could offer advantages in order to identify potential candidates for target specific manipulations and experimental studies to gain deeper insight into the pathogenic lifestyle of this emerging pathogen. With regard to empirical antibiotic therapy, no general validity for the species *W. chitiniclastica* can yet be derived from this study. With increasing numbers of available strains, preferably from different regions, but especially with a clear medical history, the analysis should be repeated and even extended to confirm and/or re-evaluate the results. Overall, our results provide the first overview of the genetic mechanisms and AMR profile of *W. chitiniclastica* that has never been presented in this form, laying the foundation for the best possible therapy.

## Data Availability Statement

The datasets presented in this study can be found in online repositories. The names of the repository/repositories and accession number(s) can be found in the article/**Supplementary Material**.

## Ethics Statement

The study was approved by the Ethics Committee at the Technical University of Dresden (EK 61022019). Written informed consent was not obtained from the individual(s) for the publication of any potentially identifiable images or data included in this article.

## Author Contributions

PS had the idea and the concept for the study. AK analyzed the data, and wrote the first version of the manuscript. BB provided the bioinformatic data from the whole genome sequences. BB, TR, SC, FG, and PS contributed text passages for the manuscript. All authors contributed to the revision of the manuscript and approved the present version. All authors contributed to the article and approved the submitted version.

## Funding

This work was supported by the Federal Ministry of Education and Research, Germany (BMBF; ZIK Septomics Research Centre, Translational Septomics, award no. 03Z22JN12 to SC).

## Conflict of Interest

The authors declare that the research was conducted in the absence of any commercial or financial relationships that could be construed as a potential conflict of interest.

## Publisher’s Note

All claims expressed in this article are solely those of the authors and do not necessarily represent those of their affiliated organizations, or those of the publisher, the editors and the reviewers. Any product that may be evaluated in this article, or claim that may be made by its manufacturer, is not guaranteed or endorsed by the publisher.
